# Ubiquitin fusion expression and tissue-dependent targeting of hG-CSF in transgenic tobacco

**DOI:** 10.1186/1472-6750-11-91

**Published:** 2011-10-11

**Authors:** Li Tian, Samuel SM Sun

**Affiliations:** 1 School of Life Sciences, Tsinghua University, Beijing 100084, China; 2 Life Science Division, Graduate School at Shenzhen, Tsinghua University, Shenzhen 518055, China; 3 School of Life Sciences, The Chinese University of Hong Kong, Shatin, N.T., Hong Kong, China

**Keywords:** hG-CSF, plant bioreactor, recombinant protein, tobacco, ubiquitin

## Abstract

**Background:**

Human granulocyte colony-stimulating factor (hG-CSF) is an important human cytokine which has been widely used in oncology and infection protection. To satisfy clinical needs, expression of recombinant hG-CSF has been studied in several organisms, including rice cell suspension culture and transient expression in tobacco leaves, but there was no published report on its expression in stably transformed plants which can serve as a more economical expression platform with potential industrial application.

**Results:**

In this study, hG-CSF expression was investigated in transgenic tobacco leaves and seeds in which the accumulation of hG-CSF could be enhanced through fusion with ubiquitin by up to 7 fold in leaves and 2 fold in seeds, leading to an accumulation level of 2.5 mg/g total soluble protein (TSP) in leaves and 1.3 mg/g TSP in seeds, relative to hG-CSF expressed without a fusion partner. Immunoblot analysis showed that ubiquitin was processed from the final protein product, and ubiquitination was up-regulated in all transgenic plants analyzed. Driven by *CaMV *35S promoter and phaseolin signal peptide, hG-CSF was observed to be secreted into apoplast in leaves but deposited in protein storage vacuole (PSV) in seeds, indicating that targeting of the hG-CSF was tissue-dependent in transgenic tobacco. Bioactivity assay showed that hG-CSF expressed in both seeds and leaves was bioactive to support the proliferation of NFS-60 cells.

**Conclusions:**

In this study, the expression of bioactive hG-CSF in transgenic plants was improved through ubiquitin fusion strategy, demonstrating that protein expression can be enhanced in both plant leaves and seeds through fusion with ubiquitin and providing a typical case of tissue-dependent expression of recombinant protein in transgenic plants.

## Background

Human granulocyte colony-stimulating factor (hG-CSF) is a hematopoietic growth factor which plays an important role in neutrophil-based immune defense against invading bacteria and other microorganisms due to its regulatory role in the proliferation, differentiation, survival and activation of neutrophils and their precursors mechanism [1,2]. Several reports showed that hG-CSF can be used to reinforce the immune system in patients with human immunodeficiency virus (HIV), pneumonia, diabetic foot infections, leukemia and febrile neutropenia [3] and to treat cancer patients undergoing chemotherapy to alleviate the depression of white blood cell levels produced by cytotoxic therapeutic agents [4,5]. Human G-CSF is now one of the important pharmaceutical proteins in cancer treatment. To satisfy the clinical needs of hG-CSF, recombinant hG-CSF has been produced in several different organisms, such as mammalian cells, yeast and *Escherichia coli *[6-8].

In the last twenty years, many recombinant therapeutic proteins have been expressed in plant production platforms [9,10] and the potential of large-scale production of pharmaceutical proteins using plant bioreactors as efficient and economical systems has been demonstrated [11-15]. However, there are only a few reports on the expression of hG-CSF in plants. Hong *et al*. [16] expressed recombinant hG-CSF in rice cell suspension culture with a maximum yield of 2.5 mg/L after 13 days incubation, but the expression in cell culture was not stable and dropped quickly. Besides this rice cell culture example, to our knowledge, there was only one report [17] on the expression of hG-CSF in plant, in which hG-CSF was expressed in the amount of 500 mg/kg fresh tobacco leaves through a transient expression system. While transient expression of recombinant proteins has its value more in laboratory research, stable expression in transgenic plants is desirable for large-scale field production at low cost. However, low protein yield has been a persistent challenge in further development of transgenic plants as a practical recombinant protein production platform. In previous studies in an attempt to express hG-CSF in plants, we experienced only low levels of its expression (lower than 0.05% total soluble protein) in transgenic *Arabidopsis *and tobacco [18].

It has been reported that expression of recombinant proteins could be enhanced by fusion with ubiquitin in *E.coli *[19,20] and yeast [21,22] by several hundred times. During such expression, ubiquitin was cleaved accurately from the fusion protein by endogenous ubiquitin-specific proteases (Ubps) in yeast, while unprocessed fusion protein accumulated in *E. coli *due to the lack of Ubps in prokaryotes. Transgenic plants had been reported capable of processing ubiquitin from its fusion partner proteins, including β- glucuronidase, luciferase and cholera toxin B subunit, and enhancing their expression in transgenic leaves by 2 to 4 fold in yield [23-25].

Here we demonstrated the application of ubiquitin fusion strategy to substantially enhance the accumulation and yield of hG-CSF in both tobacco leaves and seeds.

## Results

### Construction of expression vectors

**Figure 1:**
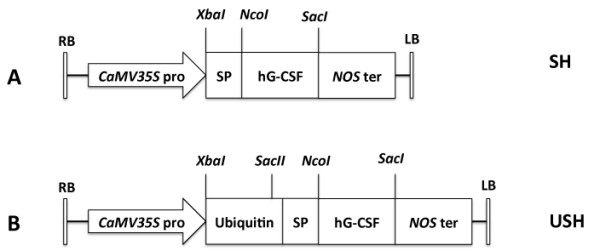
**Schematic presentation of expression cassettes**. (A) Construct SH, hG-CSF chimeric gene with phaseolin signal peptide; (B) USH, as (A) but fusion with ubiquitin. RB, right border and LB, left border of *Agrobacterium *T-DNA; *CaMV35S *pro, cauliflower mosaic virus 35S gene promoter; and *NOS *ter, nopaline synthase gene terminator.

The *CaMV *35S promoter in binary vector pBI121 was used to construct hG-CSF chimeras and the phaseolin signal peptide was introduced to direct hG-CSF into the plant cell secretory pathway. As shown in Figure 1, Construct SH, carrying the phaseolin signal peptide/hG-CSF gene, would direct the expression and entry of hG-CSF into endoplasmic reticulum (ER) for secretion and Construct USH, containing ubiquitin and signal peptide/hG-CSF genes, would allow an examination on the influence of ubiquitin fusion on the activities of Construct SH. After plant transformation and regeneration, 10 transgenic tobacco plants were obtained for Construct SH and 8 for USH.

### hG-CSF expression in leaves

**Figure 2:**
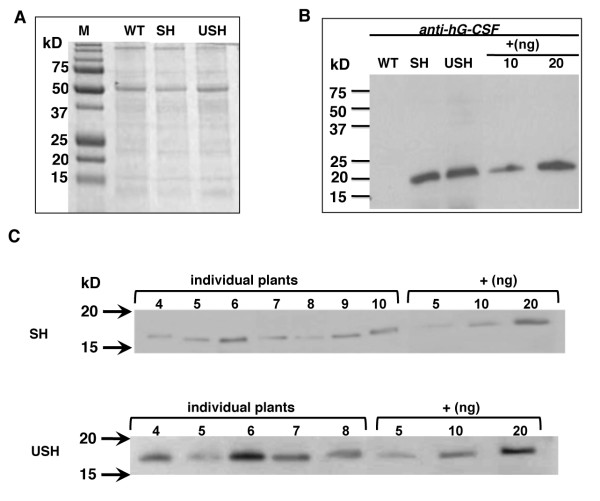
**Expression analysis of hG-CSF expressed in tobacco leaves**. (A) SDS-PAGE of total soluble protein samples (20 μg/lane) exracted from tobacco leaves. Lanes SH, USH, see Figure 1; WT, wild type tobacco; and M, Precision Plus Protein standards (Bio-Rad). (B) Immunoblot analysis of total soluble protein (20 μg/lane for plant samples, 10-20 ng/lane for comercial hG-CSF as positive control) using anti-hG-CSF antibody. (C) Expression of hG-CSF in individual SH or USH plants in addition to the plants shown in Figure 3 by immunoblot analysis. Arrows marked the location of the molecular weight at 20 and 15 kD. Numbers 4-10 in SH samples and 4-8 in USH samples denoted the individual plants analyzed. Different amounts of Comercial hG-CSF, labled by +, were loaded as postive controls at 5, 10 and 20 ng/lane accordingly.

**Figure 3:**
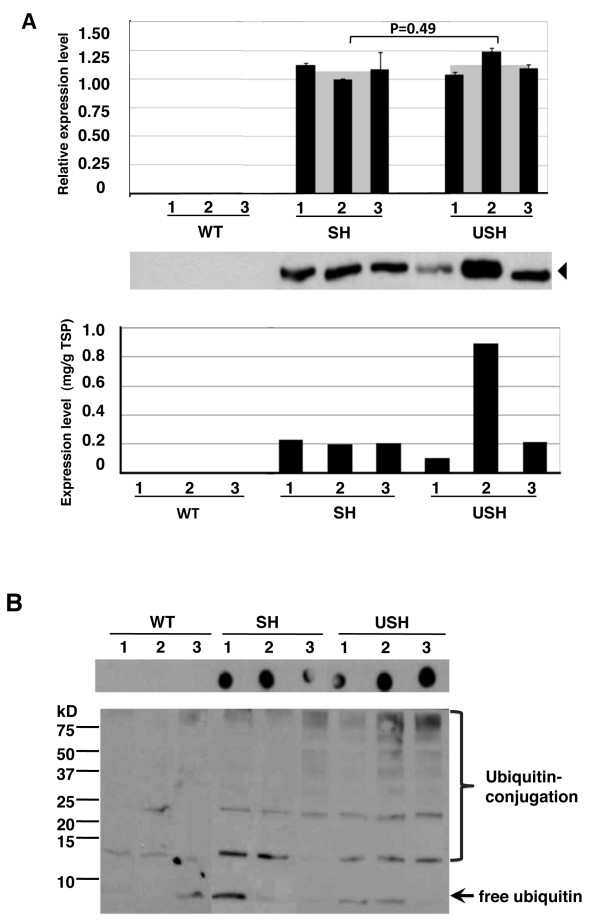
**Analysis on individual transgenic plants harboring single copy of transgene**. (A) Expression analysis of hG-CSF on individual transgenic plant leaves harboring single copy of transgene by real time PCR (upper panel) and immunoblot analysis (middle panel). Lower panel, the expression levels in individual plants determined by immunoblot as shown in middle panel. The triangle symbol marked the location of the molecular weight at 18.6 kD in accordance with comercial hG-CSF standard. All values in upper panel were normalized with 18S rRNA level and relative to the lowest expression level in SH transgenic plants. Grey squares represented the average expression of the construct based on 3 individual plants (1, 2, 3) and P values were obtained by two-tailed Student t-test on expression levels in the population of Constructs SH and USH. (B) Immunoblot analysis of endogenous ubiquitin in non-transgenic (WT) and trangenic plants using antibody against ubiquitin. Upper panel, dot immunoblot analysis of total soluble protein (2 μg/dot) in tobacco leaves, and lower panel, immunoblot analysis of total soluble protein (10 μg/lane). Free ubiquitin, a 8.5 kD protein, was indicated by arrow, and ubiquitin-cojugated proteins, including the smear high molecular weight component, were denoted by the bracket. Lanes SH and USH, see Figure 1.

The expression of hG-CSF in tobacco leaves carrying different gene constructs were detected by SDS-PAGE and immunoblot analysis (Figure 2). For total soluble protein sample, no distinct difference in protein banding patterns was observed between wild-type (WT) and transgenic plants (Figure 2A). For Construct SH, hG-CSF was synthesized with the same molecular weight (MW) of 18.6 kD as commercially available hG-CSF produced in *E.coli *(Figure 2B). As to Construct USH, i.e. hG-CSF in fusion with ubiquitin, if ubiquitin could be cleaved, the signal peptide would direct the expressed hG-CSF polypeptide into ER and the final product would have a MW of 18.6 kD as SH transgenic plants; if not, the fusion protein would remain intact with a MW about 30 kD. The results of immunoblot analysis (Figure 2B) showed that the synthesized hG-CSF was of 18.6 kD, indicating both ubiquitin and signal peptide were absent in the final protein product. Because the removal of signal peptide generally occurs at ER while ubiquitin in cytosol, it appears that ubiquitin was processed before the nascent protein entering into the ER. When ubiquitin antibody was used to detect the total soluble proteins in SH and USH plants, no protein band of similar molecular weight (18.6 kD) was detected (Figure 3B), confirming that ubiquitin was not present in the final product.

The accumulation levels of hG-CSF in leaves were estimated by immunoblot through comparison with known amounts of commercial hG-CSF (Figure 2C, Figure 3A and Table 1). On average, the level was about 0.3 mg/g total soluble protein (TSP) in SH transgenic plants while 0.89 mg/g TSP in USH transgenic plants. When individual plants were considered, the maximum accumulation of hG-CSF in transgenic USH leaves could reach 2.58 mg/g TSP, 7 fold higher than the 0.38 mg/g in SH transgenic plants, suggesting that ubiquitin fusion did enhance the accumulation of hG-CSF significantly in transgenic tobacco leaves.

**Table 1:** Accumulation of hG-CSF in tobacco leaves and seeds

Tissue	Copy number^1^	Construct	Number of plants^2^	Average Yield^3^*	Maxium Yield^4^*
Leaf	single	SH	3	0.21 ± 0.01	0.23
		USH	3	0.42 ± 0.40	0.89
	_	SH	10	0.30 ± 0.07	0.38
		USH	8	0.89 ± 0.77	2.58
Seed	single	SH	3	0.45 ± 0.11	0.53
		USH	3	0.93 ± 0.12	1.07
	_	SH	10	0.50 ± 0.15	0.77
		USH	8	0.97 ± 0.19	1.38

^1^, Copy number of trangene determined by Southern blot: single, 1 copy of transgene; -, without consideration to transgene copy number.

^2^, Number of individual transgenic plants anlyzed for each tissue, construct and different copy numbers of transgene.

^3^, Values are the means ± stand deviation (SD).

^4^, Maxium yield among transgenic plants anlyzed for each construct and tissue.

*, mg/g TSP for leaf and seed, estimated by quatitative analysis of immunoblot data with ImageJ software.

### Analysis on transgenic tobacco leaves carrying a single transgene

To investigate the impact from ubiquitin fusion on the expression of hG-CSF, three individual plants carrying a single copy of target gene (based on Southern blot analysis, data not shown) were selected from SH and USH transgenic plants, respectively, and real-time PCR (RT-PCR) was used to quantify the relative expression of hG-CSF at steady-state mRNA level. As shown in Figure 3A, upper panel, the relative expression of hG-CSF at transcription level in SH and USH transgenic plants showed no significant differences. However, the expression of hG-CSF at protein level in leaves of these plants displayed marked differences (Figure 3A, middle and lower panels) and the accumulation levels on average were 0.21 mg/g TSP for SH plants and 0.42 mg/g TSP for USH plants while the maximum level was about 0.23 mg/g TSP for SH and 0.89 mg/g for USH, suggesting that even among the transgenic plants carrying single transgene, ubiquitin fusion could still notably enhance the accumulation of hG-CSF in transgenic tobacco leaves.

To examine the transgene effect on endogenous ubiquitin and ubiquitination in transgenic plants, dot immunoblot analysis of total soluble protein from leaves was performed using antibody against plant ubiquitin (Figure 3B). At the level of 2 μg/dot of protein and film exposure time of 2 minutes during immunoblotting, clear signals were detected for all samples transformed with Construct SH or USH while no positive signal was detected in WT plants, suggesting that ubiquitination of transgenic plants might be up-regulated. To confirm the results of dot immunoblot analysis, SDS-PAGE of the total soluble protein (10 μg/lane) followed by immunoblot analysis were performed. As shown in Figure 3B, after the same exposure time, notable smear protein patterns (ubiquitin-conjugated proteins) were observed in the leaves of SH and USH transgenic plants, but fewer bands in the WT plants. These results indicated that ubiquitination was up-regulated in transgenic plants.

### hG-CSF expression in seeds

**Figure 4:**
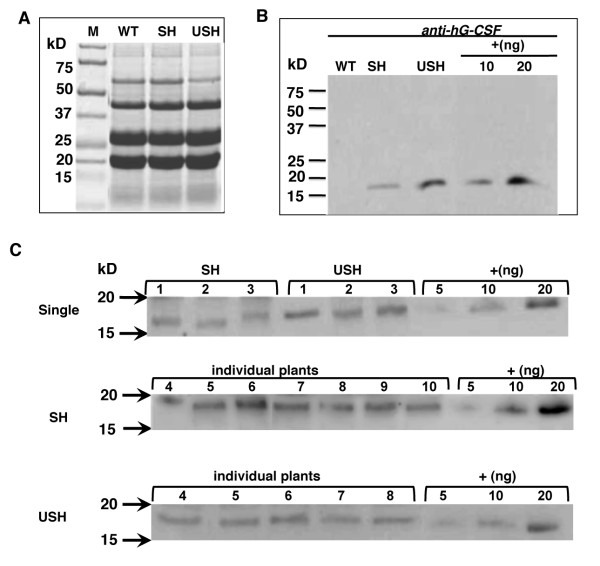
**Expression analysis of hG-CSF in transgenic tobacco seeds**. (A) SDS-PAGE analysis of total soluble protein (50 μg/lane) extracted from tobacco seeds. Lanes SH, USH, see Figure 1; WT, wild type tobacco; and M, Precision Plus Protein standards (Bio-Rad). (B) Immunoblot analysis of total soluble protein (20 μg/lane for sample proteins and 10-20 ng/lane for comercial hG-CSF as positive control) using anti-hG-CSF antibody. (C) Expression of hG-CSF in individual SH or USH plants as detected by immunoblot analysis. Single, immunoblot analysis of hG-CSF of individual transgenic plants harboring single copy of transgene. Arrows marked the location of the molecular weight at 20 and 15 kD. Numbers 4-10 in SH samples and 4-8 in USH samples denoted the individual plants analyzed. Different amounts of Comercial hG-CSF, labled by +, were loaded as postive controls at 5, 10 and 20 ng/lane accordingly.

The expression of hG-CSF in transgenic tobacco seeds was analyzed by similar methods as in leaves. The expression of hG-CSF in tobacco seeds harboring Construct SH or USH was detected by SDS-PAGE and immunoblot analysis (Figure 4). For total soluble protein samples, no distinct difference in protein banding patterns was observed between WT and transgenic plants by SDS-PAGE analysis (Figure 4A), and immunoblot analysis showed that in both SH and USH transgenic seeds, hG-CSF was synthesized with the same MW of 18.6 kD as commercially available hG-CSF produced in *E.coli *(Figure 4B), suggesting that both ubiquitin and signal peptide were absent in the final protein product.

As shown in Figure 4C and Table 1 the accumulation level of hG-CSF protein, on average, was 0.5 mg/g total soluble protein (TSP) in SH transgenic seeds while 0.97 mg/g TSP in USH, a 2 fold over the SH seeds, and the highest accumulation of hG-CSF was 0.77 mg/g TSP in SH and 1.38 mg/g TSP in USH transgenic seeds, respectively, indicating that ubiquitin fusion could also be applied to enhance the accumulation of hG-CSF in seeds. When single copy of transgene was considered, the accumulation levels on average were 0.45 mg/g TSP for SH plants and 0.93 mg/g TSP for USH plants, suggesting that among the transgenic plants carrying single copy of transgene, ubiquitin fusion could still enhance the accumulation of hG-CSF in transgenic seeds. A summary on the expression of hG-CSF in transgenic tobacco was shown in Table 1.

### Intracellular localization of hG-CSF

**Figure 5:**
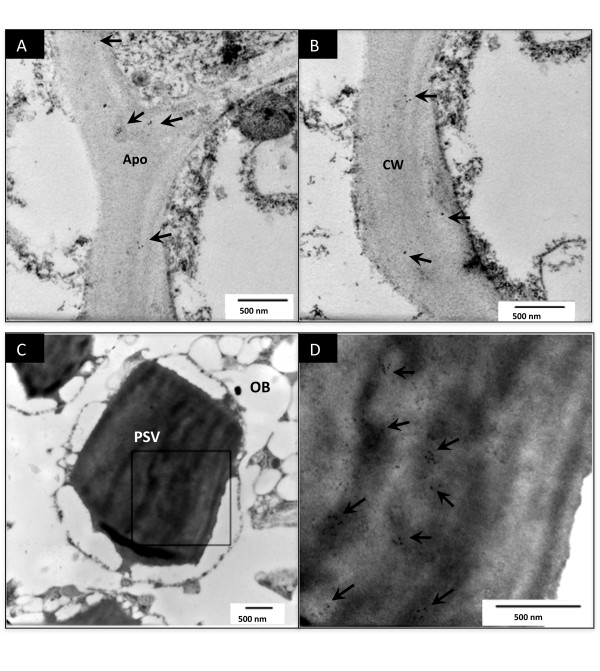
**Subcellular localization of hG-CSF in transgenic tobacco leaves and seeds**. The leaf (A, B) and seed (C, D) samples from the same transgenic tobacoo plant harboring Construct USH was analyzed by immunoelectron microscopy using anti-hG-CSF antibody. Arrow indicated immuno-labeling of hG-CSF. Panel D represented a higher magnification of the rectangle area in panel C. Apo, apoplast; CW, cell wall; PSV, protein storage vacuole; and OB, oil body.

Based on the information of the two constructs as shown in Figure 1, with no other targeting peptide, except the phaseolin signal peptide introduced to the hG-CSF, it is likely that the expressed hG-CSF in plant cells should be secreted via the default pathway. The intracellular localizations of hG-CSF in leaf and seed from the same transgenic plant carrying Construct USH were analyzed by immuno-electron microscopy. The target hG-CSF was found to appear in the apoplast and cell wall of leaf (Figure 5A and 5B). However, for transgenic tobacco seeds, hG-CSF was observed in protein storage vacuoles (PSV) (Figure 5C and 5D) while no positive labeling in apoplast or other cell compartments was detected (data not shown).

### Bioactivity assay

Bioactivity analyses of hG-CSF expressed in transgenic leaves and seeds were performed by measuring their capability to promote the proliferation of hG-CSF-dependent NFS-60 cell line which grow only under the presence of hG-CSF or other known growth factors [26]. Total soluble protein samples (TSP) containing 1 ng of recombinant hG-CSF (determined by immunoblot, data not shown) extracted from USH leaves and seeds were used for bioactivity assay. For comparison, protein samples from transgenic SH or WT leaves and seeds, containing the same amount of TSP as in USH samples, were also included. To exclude potential influence from transgenic effect, total soluble protein samples from leaves and seeds of transgenic tobacco carrying empty pBI121 plasmid (denoted as EP) were used as negative controls while commercial hG-CSF produced by *E.coli *was used as positive control.

**Figure 6:**
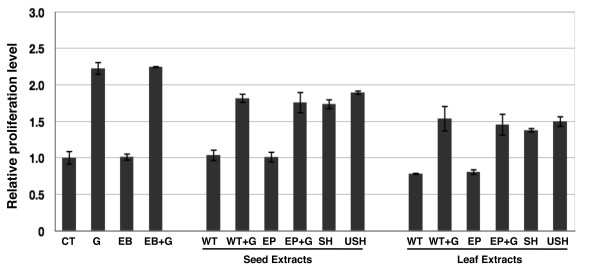
**Bioactivity analysis of the expressed hG-CSF in transgenic leaves and seeds**. Total soluble protein (TSP) extracted from leaves (leaf extracts) and seeds (seed extracts) was analyzed. CT, untreated sample; G, samples treated with 1 ng comercial hG-CSF; EB, extraction buffer; EB+G, extraction buffer supplemented with 1 ng comercial hG-CSF; WT, SH and USH, TSP from WT, SH and USH leaves and seeds; EP, TSP from transgenic plant seeds harboring empty pBI121 vector; WT+G, WT sample supplemented with 1 ng comercial hG-CSF; EP+G, EP sample supplemented with 1 ng comercial hG-CSF. Number in Y axis represented the raltive value to untreated sample (CT).

As shown in Figure 6, after 72 hours incubation, the cells treated with extraction buffer (sample EB) showed similar baseline proliferation level with the untreated sample (sample CT). When treated with commercial hG-CSF (sample G), the proliferation of NFS-60 cells was promoted by 220% over the untreated sample CT while sample EB+G (extraction buffer supplemented with hG-CSF) showed similar results, confirming that no influence was from the extraction buffer and the validity of the analysis system.

In the analysis of seed extracts, the cells treated with samples WT and EP (transgenic plant carrying empty PBI121 plasmid) showed similar proliferation levels as the untreated (CT), suggesting that no distinct background effect from tobacco seed proteins and transgene process. Compared with the samples WT and EP, USH protein sample containing 1 ng expressed hG-CSF could promote the proliferation of NFS-60 cells, indicating that the expressed hG-CSF in USH seeds was bioactive. SH protein sample containing relatively less (0.9 ng) hG-CSF also showed biological activity but with corresponding decrease in cell proliferation activity. However, the bioactivity of USH sample was lower than those of samples G and EB+G which contained the same amount of hG-CSF while similar results were also observed with samples WT+G and EP+G, suggesting that some compounds in tobacco seeds may influence the efficacy of hG-CSF.

In the analysis of leaf extracts, proliferation effect of NFS-60 cells by USH and SH leaf protein samples were also observed, suggesting that the expressed hG-CSF in leaves was also in bioactive form. A notable difference was that the total soluble protein from tobacco leaves, in comparison to that from seeds, appeared to show some inhibitory effect on the growth of NFS-60 cells, as the cells treated with leaf extracts, with or without hG-CSF, yielded lower proliferation activity than the seed extracts and the control group. Further, protein samples from old leaves and leaves after freezing-thawing treatment appeared to exert harmful effect on the growth of NFS-60 cells even with the addition of commercial hG-CSF (see Additional file 1), suggesting that some compounds extracted from tobacco leaves were harmful to the growth of NFS-60 cells or influenced the function of culture medium.

## Discussion

Many recombinant therapeutic proteins have been expressed in plant-based bioreactor, but only a few studies were on the expression of hG-CSF which is widely used in clinical treatments. In this article, we reported the expression of hG-CSF in transgenic tobacco leaves and seeds. To improve the expression of recombinant proteins in *E. coli *and yeast, ubiquitin fusion strategy has been used and shown to significantly enhance their expression [19-22]. However, only a few reports were on the use of ubiquitin fusion strategy in transgenic plant leaves [23-25] but not in seeds. Through the strategy of ubiquitin fusion, our study demonstrated that the accumulation of hG-CSF could be increased up to 2 to 7 fold in leaves and 2 fold in seeds relative to unfused hG-CSF expression, suggesting that the expression enhancement is applicable to plant seeds as well and providing the first evidence on using the ubiquitin fusion strategy to enhance the expression of a target protein in seeds which are considered as bioreactor platform with distinct advantages [15].

Expression of recombinant hG-CSF was studied in rice and tobacco cell suspension cultures and tobacco leaves through transient expression system but not in transgenic plants (see Additional file 2). It's known that transient expression often lead to high accumulation of target recombinant proteins favoring laboratory study. However, for scale-up production of plant-based pharmaceuticals, transgenic plant is a better choice. This study provides the first proof on the expression of bioactive recombinant hG-CSF in transgenic plants, and that the production can be augmented by ubiquitin fusion strategy, with a maximum accumulation level reaching 2.5 mg/g (0.25%) total soluble protein (TSP) in leaves and 1.3 mg/g (0.13%) TSP in seeds. However, these levels of expression, though notably improved in comparison to other reports (see Additional file 2), are still in low range. The expression of another valuable human colony stimulating factor (CSF), human granulocyte-macrophage colony stimulating factor (hGM-CSF), widely used as an adjuvant in clinical treatment of neutropenia and aplastic anemia [27], has been reported by several studies (see Additional file 2). Through a survey on the transgenic expression of CSF in plants (see Additional file 2), except the high expression levels through transient expression system and a case in rice seed, it seems that human colony stimulating factor is difficult to be expressed at levels higher than 0.1% TSP, possibly resulting from its poor-efficient translation in plants due to unfavorable codon usage and/or instability of the expressed human CSF in plant cell environments. Thus, further investigations are necessary for improvement.

The native hG-CSF is a glycoprotein with an O-glycosylation site at residue Thr_133 _and the MW of hG-CSF is about 18.6 kD without glycosylation as shown in Figures 2 and 4 (positive controls, produced by *E. coli*) but varied from 17 to 24 kD when produced in plant-based systems (see Additional file 2). The expressed hG-CSF in tobacco leaves and seeds in this study showed a MW around 18.6 kD as the unglycosylated hG-CSF. Whether partial or complete glycosylation occurred couldn't be concluded as the glycosylation patterns may vary among different organisms, tissues and even different cells. In this connection, potential immunogenicity of recombinant proteins as a result of non-human glycosylation should also be considered in the application of plant bioreactors. This aspect of concern has been reviewed in several published review papers [9,13,14,28]. To tackle the problem on N-glycosylation, many efforts were made to control the glycosylation of recombinant proteins in plants, such as knock-in or knock-out of related enzymes and retention of the target protein in ER [13,28]. We also suggested some different strategies on using membrane anchors for delivering recombinant proteins via or bypass Golgi transport pathway in control of glycosylation [14]. Only little attention has been paid to the O-glycosylation status of therapeutic proteins produced in transgenic plants thus far, although marked differences in the O-glycosylation machinery exist between humans and plants [28]. It was anticipated that the presence of plant-modified O-glycans would lead to accelerated clearance of the glycoprotein from the blood [28]. However, no direct proof could be provided at present time. In the case of hG-CSF, no N-glycosylation site could be predicted and thus the glycosylation is likely to come from O-linked modification.

However, immunogenicity of O-linked glycans is presently unknown. Further, the glycosylation of hG-CSF is not essential for its bioactivity in terms of clinical effect [29,30], and the bioactivity assay showed that the expressed hG-CSF possessed *in vitro *biological activity in inducing the proliferation of NFS-60 cells, indicating that hG-CSF produced in both tobacco leaves and seeds was in bioactive form and ubiquitin fusion expression didn't affect its bioactivity.

There are a total of five cysteine residues in hG-CSF protein and two intra-chain disulfide bonds are formed between Cys_36 _and Cys_42 _and between Cys_64 _and Cys_74 _for proper folding of the protein [31]. The free cysteine residue in mature hG-CSF protein may link with the free cysteine residue of another hG-CSF protein through disulfide bonding to form a dimer. In this study, dimerization of hG-CSF protein through disulfide bond formation was indeed observed in transgenic seeds, but no dimerization was detectable in leaves (see Additional file 3), suggesting that the environment of seed organ favors recombinant hG-CSF to form dimer. Although it has been reported that hG-CSF functions as monovalent ligand and hG-CSF dimers showed lower *in vitro *activity than the monomers [32], the recombinant hG-CSF produced in seeds showed similar biological activity as the commercial hG-CSF presented in WT protein extract in this study, which in turn suggested the occurrence of proper folding of the expressed hG-CSF.

On the enhancement of protein expression through fusion with ubiquitin, several mechanisms were proposed: (i) N-end protection [20,25] - Rapid folding of the nascent ubiquitin moiety at the N-terminus of an emerging polypeptide chain may protect the still unfolded chain from co-translational degradation; (ii) Folding facilitation [33,34] - It was suggested that expression of ubiquitin fusion would enhance the solubility of recombinant proteins in *E.coli*, and ubiquitin moiety at the N-terminus facilitated the folding of the emerging protein; and (iii) Efficient translation [20,25] - Ubiquitin is an extremely conserved protein, and the favorable codon bias of ubiquitin enhances translation of the appended coding region. However, there is no sufficient evidence to support these proposed explanations and the enhancement mechanism by ubiquitin fusion remains unclear. In our study, RT-PCR results indicated that there was no significant difference in the level of steady-state mRNA between fusion of hG-CSF with ubiquitin or not. The difference observed in expression between fusion to ubiquitin (USH) and unfused (SH) does not appear to be affected at the transcriptional level, and therefore differences in expression levels are post-transcriptionally affected, perhaps at translational or post-translational level. This hypothesis is based on the obervation that when hG-CSF was expressed without targeting through the secretory pathway (no signal peptide) in tobacco, the recombinant protein was not detected (see Additional file 4, Construct H). This may suggest that the recombinant protein was readily degraded when expressed in the cytosol and failed to accumulate; however when fused to ubiquitin hG-CSF was detected (see Additional file 4, Construct UH). Together, this supports the notion that ubiquitin moiety at the N-terminus may protect the protein from proteolytic attack in transgenic plants. However, it remains possible that ubiquitin facilitates folding and efficient translation, which in turn contribute to the enhancement.

Ubiquitin processing from the initial translation product was shown to be so efficient that unprocessed product couldn't be detected and such processing has been observed in several plants including tobacco, rice and potato [25]. In this study, transgenic plants carrying Construct USH could lead to the expression of hG-CSF without ubiquitin in the final product, further supporting the efficient processing of ubiquitin in tobacco leaves and seeds. It has been indicated that ubiquitin is processed from the translation product by Ubps co-translationally and the efficient and rapid cleavage of ubiquitin guarantees the docking of signal peptide with the ER and protein import into the ER [25,35]. These molecular events are supported by our study in that the expressed hG-CSF was imported into the ER and further transported to apoplast in leaves and protein storage vacuoles in seeds. Although we did not determine the N-terminal sequence of the expressed hG-CSF in tobacco, ubiquitin cleavage site has been previously demonstrated by studies to occur precisely after the final amino acid glycine, residue G-76, in the C-terminus of ubiquitin [25,36,37]. Using similar cloning procedure and chimeric gene constructs (see Methods), we expect that the ubiquitin fusion strategy will direct the production of recombinant hG-CSF without change in its amino acid sequence at the N-terminus.

Transgenic plants have been developed for many years, but the possible endogenous changes in transgenic plants that may bring about by the effects of transgenes have not been fully studied. Luo *et al*. reported that more proteins were degraded by ubiquitin-proteasome pathway in the transgenic endosperm when human granulocyte-macrophage colony stimulation factor (hGM-CSF) was expressed in rice cells [38]. This consideration was based on the observation of up-regulation of ubiqutinated proteins which might lead to possible ubiquitination and degradation of hGM-CSF through the ubiquitin-proteasome pathway. Our study also showed similar up-regulation of protein ubiquitination in transgenic plants, suggesting the possible occurrence of more ubiquitin-dependent protein degradation events. However, in our case, no ubiquitinated hG-CSF was detected in transgenic plants harboring the Construct H (see Additional file 4) by immuno-precipitation and immunoblot assay (data not shown), so the degradation pathway of recombinant hG-CSF in transgenic plants can not be asserted. Interestingly, ubiquitin fusion led to variable expression of the hG-CSF in individual plants even those with single copy of transgene (Figure 3A, Construct USH) while the plants without ubiquitin fusion showed relatively consistent expression (Figure 3A, Construct SH). Because ubiquitin is an important protein functioning in many different aspects of all eukaryotic cells [39], the additional ubiquitin together with the transgene effects may result in broader impact on the endogenous environment of transgenic plants than non-fusion transgenic plants, which in turn may lead to variations at the level of hG-CSF expression under ubiquitin fusion.

Studies on the intracellular location of recombinant proteins offer helpful hints in choosing a strategy on their expression and downstream purification in the development of plant bioreactor. In this study, using a constitutively expression promoter, the protein of interest was proposed to be secreted via the default pathway because no particular sequence information was provided for specific transport of the recombinant hG-CSF. However, while hG-CSF was found to be secreted outside the cell in leaves, it appeared only in the PSV of seeds. Based on the sequence of hG-CSF, no targeting peptide could be predicted. It thus appears that tissue-dependent localization of hG-CSF in transgenic tobacco happened in our case. There were some reports on tissue or cell-specific deposition of exogenous proteins in plants, such as the secretion of phytohemagglutinin, a PSV resident seed protein, into the apoplast of root tissues of the common bean (*Phaseolus vulgaris*) [40] and the accumulation of pathogenesis-related protein, normally secreted by other cell types, in the vacuoles of specialized cells called crystal idioblasts [41]. These examples indicate that protein targeting may be regulated in a tissue- or cell-specific manner [42], and recombinant proteins expressed in seeds may be deposited in unexpected places [42-46]. To compare intracellular trafficking in different tissues, Drakakaki *et al*. [42] used *Aspergillus niger *phytase as a model glycoprotein to study the intracellular fate of recombinant protein in the leaves, calli and seeds of rice and found that the recombinant protein was efficiently secreted from leaf cells and calli as expected, but in contrast, it was retained in the endoplasmic reticulum (ER)-derived prolamin bodies and protein storage vacuoles in the endosperm cells. In the study, however, the recombinant protein expression was driven by different promoters, i.e. *CaMV *35S promoter for leaves and calli while Gt1 promoter for seeds. In our study, driven by the *CaMV *35S promoter only, hG-CSF produced in transgenic tobacco was observed in apoplast in leaves but in PSV in seeds, providing a more typical case of tissue-specific deposition of an exogenous protein in transgenic plant.

Although our and previous studies indicated that recombinant proteins could be accumulated into unexpected compartments in transgenic seeds instead of via secretory pathway [42-46], there were still some notable inverse situations. Leite *et al*. [47] reported that a recombinant human growth hormone, driven by a seed-specific promoter from sorghum γ -kafirin seed storage protein gene with a signal peptide from a *Coix *prolamin, was secreted into the apoplastic space in transgenic tobacco seeds. Therefore, tissue-specific protein sorting appears to be a complicated process, and further research is needed to elucidate the underlying mechanisms, such as the effects of promoters and individual proteins.

The bioactivity analyses on total soluble protein from tobacco leaves and seeds were also performed in this study. Results revealed that compared with seed extracts, total soluble protein from leaves had lower promotional activity on the proliferation of NFS-60 cells, suggesting that the source of tissues might affect the bioactivity of the product. Although total soluble protein extracted from seeds didn't exert adverse effect on the growth of NFS-60 cells, it did somewhat influence the efficacy of hG-CSF, suggesting that some endogenous proteins or compounds in the host plants may react with the target protein to inhibit its bioactivity. Total protein samples from young leaves had minor while old or degraded leaves (such as after repeated freezing and thawing) exerted greater inhibitory effect on the growth of NFS-60 cells, suggesting that developmental and physiological status and conditions of the plant tissues may also affect the bioactivity of the products. In summary, the selection of host plants, the endogenous chemical and biological environments of specific organs or tissues and their storage conditions should be considered when transgenic plants are used to produce therapeutic proteins. Thus proper stage of tissue collection, strict tissue storage conditions, efficient extraction and purification steps to obtain material free of endogenous host cell proteins/components are critical in achieving desired efficacy and safety of the therapeutic.

## Conclusions

In this study, the expression of bioactive hG-CSF, an important human cytokine widely used in clinical treatment, was enhanced through the strategy of ubiquitin fusion in both transgenic tobacco leaves and seeds, providing the first evidence of the expression of hG-CSF in a stably transformed plant and the applicability of the ubiquitin fusion strategy to improve recombinant protein expression in transgenic plant seeds. Tissue-dependent targeting of recombinant hG-CSF was also observed in the study, suggesting that protein sorting may be affected by a tissue-specific mechanism and subcellular localization should be considered in designing recombinant protein production in different tissues of transgenic plants.

## Methods

### Construction of expression vectors

Ubiquitin coding sequence was cloned from genomic DNA of tobacco leaves using primers u-f (5'-GCTCTAGAATGCAGATCTTCGTCAAAACCCTC-3') and u-r (5'-GCGAGCTCACCACCGCGGAGACGGAG-3') designed based on the tobacco ubiquitin gene [GenBank: AJ309010], in which a SacII site (underlined) was introduced 10 bp upstream from the teriminal G codon [25] without any shift and change in the open reading frame. The hG-CSF gene was donoted by Dr. Wei Han (The Memorial Sloan-Kettering Cancer Center, New York). DNA encoding hG-CSF with phaseolin signal peptide was cloned into pBI121 binary vector as shown in Figure 1, resulting in Construct SH. Phaseolin signal peptide was used to direct the expressed hG-CSF into ER. To guarantee no additional amino acid introduced to N-terminus of signal peptide and hG-CSF, DNA encoding hG-CSF with phaseolin signal peptide was modified to include an appended DNA sequence of the downstream 16 bp of ubiquitin gene, CACCGCGGAGACGGAG, and cloned into pBI121 binary vector with ubiquitin sequence through the introduced SacII site (underlined), resulting in Construct USH (Figure 1). All the expression cassettes were driven by the cauliflower mosaic virus (*CaMV*) 35S promoter and terminated by the nopaline synthase (NOS) terminator.

### *Agrobacterium*-mediated transformation

All chimeric genes in pBI121 expression vectors were transformed into *Agrobacterium tumefaciens *LBA4404 by electroporation. Young leaves from wild type tobacco were cut into small square discs (0.5 × 0.5 cm^2^) and immersed in 10× diluted agrobacterial culture for 10 minutes. The leaf discs were first transferred to sterile filter paper for excess agrobacterium removal and then transferred onto solidified co-cultivation MS medium (Sigma). After co-cultivation, the transformants were selected on MS medium containing 500 mg/L carbenicillin (Sigma) and 100 mg/L kanamycin (Sigma). Regenerated tobacco plants were transferred to soil finally and grown in green house (The Chinese University of Hong Kong) to maturity. Positive transformants were identified by PCR screening and Southern blot analysis of genomic DNA.

### RNA extraction and Real-time PCR

Total RNA was extracted using Tripure Isolation Reagent (Roche) from the young fresh leaves of 21-day-old transgenic plants and purified with RNeasy Plant Mini Kit (Qiagen). Total RNA samples was diluted with RNase-free water, and stored in -80°C ultra low temperature refrigerator. RNA concentration was determined by OD_260 _measurement using a spectrophotometer and the quality of RNA was checked by 1% agarose/formaldehyde gel electrophoresis.

The level of hG-CSF transcript was quantified by two-step real-time PCR. Complementary cDNA synthesis was conducted following the kit protocol for Powerscript Reverse Transcriptase (Clontech) using Oligo-dT primer (Promega). For hG-CSF amplification, cDNA product from 100 ng total RNA was used as template in each RT-PCR reaction with h-f (5'- CCACCCCCCTGGGCCCT-3') and h-r (5'-GGGCTGGGCAAGGTGGC-3') primers, and 10 ng total RNA for *18 S *rRNA [GenBank: AJ236016] as internal control with 18 s-f (5'-AGGAATTGACGGAAGGGCA-3') and 18 s-r (5'-GTGCGGCCCAGAACATCT-3') primers. RT-PCRs were performed using the Bio-Rad iQ5 Real-Time PCR System with SYBR Green Supermix Kit (Bio-Rad). All samples, including the external standards and non-template control, were run in triplicate. The reaction was initiated by activation of Taq polymerase at 95°C for 5 min, followed by 40 three-step amplification cycles consisting of 30 s denaturation at 95°C, 30 s anealing at 60°C and 45 s extension at 72°C. The fluorescence signal was measured at the end of each extension step at 72°C. A final dissociation stage was run to generate a melting curve for verification of amplification product specificity. Following the final PCR cycle, the reactions were heat-denatured at 0.5°C/10 s from 55 to 95°C. Data analysis was performed by Gene Expression Function on the Bio-Rad iQ5 software.

### Protein extraction and Immunoblot

#### Leaf

Total soluble protein was extracted from fresh young leaves of 21-day-old transgenic plants with 50 mM phosphate buffered saline (PBS) extraction buffer in the presence of complete protease inhibitor (cocktail tablets, Roche). Fresh leaves (1 g) were ground into powder in a mortar with liquid nitrogen and 1 ml extraction buffer was added in. Whole homogenate was transferred into 2 ml Eppendorf tube, incubated on ice for 15 min and centrifuged at 20000 × g for 10 minutes. Supernatant was collected and centrifuged for another 10 minutes. Final supernatant was collected as total soluble protein (TSP) from leaves. Protein concentration was determined by BCA assay (Pierce) with bovine serum albumin (Sigma) as standard.

#### Seed

Mature seeds were used to extract the recombinant protein. Seeds (10 mg) were ground into powder in a mortar with 200 μl 50 mM phosphate buffered saline (PBS) extraction buffer in the presence of complete protease inhibitor (cocktail tablets, Roche) and sonicated for 30 minutes in ice water. The remained steps were as those of the leaf procedure.

#### Immunoblot/SDS-PAGE

Total soluble protein extracted from leaf or seed was diluted with 4× loading buffer (0.2 M Tris-HCl, pH 6.8; 0.8 g SDS; 40% Glycerol; 5%β-mercaptoethanol; 50 mM EDTA; 8 mg Bromophenol Blue), boiled for 5 minutes and separated on 15% SDS-PAGE with 10-50 μg protein/lane followed by Coomassie Brilliant Blue Staining for protein visualization. For immunoblot, the total protein separated by SDS-PAGE was directly transferred to PVDF membrane without staining. To test on the formation of dimers by recombinant hG-CSF, protein samples were treated with 4× loading buffer without β-mercaptoethanol as controls. The immunoblotting was carried out using rabbit polyclonal anti-hG-CSF antibody (PeproTech) as primary antibody and anti-rabbit IgG-Peroxidase antibody (Sigma) as secondary antibody and developed using the ECL detection system (Amersham Co., Bucks, UK). Recombinant hG-CSF from *E.coli *was purchased from PeproTech and used as positive control. In the immunoblot analysis of ubiquitin and ubiquitin-conjugated proteins, rabbit polyclonal anti-ubiquitin antibody (specific to plant ubiquitin, Novus Biologicals) was used as primary antibody. To estimate the expression level of hG-CSF in transgenic plants, the same amount of total soluble protein from leaves or seeds was loaded onto SDS-PAGE during immunoblotting while leaving 3 lanes for positive control standards of commercial hG-CSF at 5, 10 and 20 ng/lane, respectively. The detected immunoactive bands of the experimental samples were compared with the positive controls and quantified by densitometry using the ImageJ software (National Institute of Health, USA; http://rsbweb.nih.gov/ij/). All the quantity of samples fell within the amount of the three control standards.

### Bioactivity Assay

The proliferation assay of NFS-60 cells in response to the presence of hG-CSF measured by MTT [3-(4,5-dimethylthiazol-2-yl)-2,5-diphenyl tetrazolium bromide] method was used to test the bioactivity of expressed hG-CSF in plants. NFS-60 cells were cultured with RPMI-1640 medium (Gibco) supplemented with 10% fatal bovine serum (FBS) and commercial recombinant hG-CSF (2 ng/ml, PeproTech) and kept under 5% CO_2 _at 37 °C in a humidified condition. For MTT test, total soluble protein extracted from leaves and seeds (see Protein extraction and immunoblot above) were used for analysis and NFS-60 cells (1 × 10^4 ^per well) were cultured in a 96 well plate and treated with the medium (RPMI-1640 medium supplemented with 10% FBS) containing the following samples: commercial recombinant hG-CSF (1 ng); total soluble protein extracted from leaf (TSP-L) and seed (TSP-S) of transgenic plant harboring the Construct USH containing 1 ng expressed hG-CSF; TSP-L and TSP-S from SH plant, containing the same amount of total protein as the USH samples while 0.8 and 0.9 ng expressed hG-CSF, respectively; TSP-L and TSP-S from transgenic plant harboring empty pBI121 plasmid (denoted as EP), containing the same amount of total protein as the USH samples; TSP-L and TSP-S from EP plant (as above) supplemented with 1 ng commercial hG-CSF; TSP-L and TSP-S from wildtype (WT) plants, containing the same amount of total protein as the USH samples; TSP-L and TSP-S fromWT plant (as above) supplemented with 1 ng commercial hG-CSF; extraction buffer (EB, PBS in supplement with protein inhibitor); extraction buffer supplemented with 1 ng commercial hG-CSF. All treatments, including the untreated control, were carried out in triplicate. Cells were incubated at 37 °C for 72 hours. Finally, 20 μl of MTT (5 mg/ml) was added and the samples were left to incubate at 37 °C for 4 h. The medium was discarded and the formazan dye was dissolved in DMSO (100 μl) at 37 °C for 30 min. Absorbance was measured for all samples at 550 nm using a VICTOR3 V™ Multilabel Counter (PerkinElmer, USA).

### Immunohistochemistry

#### Leaf

Immunogold electron microscopy (EM) on ultrathin sections prepared from fresh young leaves was performed as previously described [48]. Small discs of tobacco leaves were frozen in a high-pressure freezing apparatus (Leica EM PACT2). Substitution was performed in an AFS freeze substitution unit (Leica). Samples were stepwise infiltrated, embedded, and UV polymerized with Lowicryl HM20 (Electron Microscopy Sciences). Immunolabeling on HM20 ultrathin sections was done using standard procedures with hG-CSF antibodies at 1:50 dilution, and gold-coupled secondary antibodies at 1:50. Aqueous uranyl acetate/lead citrate post-stained sections were examined in Hitachi H-7650 transmission EM with a CCD camera (Hitachi High-Tech, http://www.hitachi-hitec.com) operating at 80 kV.

#### Seed

Immature seeds were collected at 14-16 days after flowering and fixed at 4°C for overnight in 4%(v/v) paraformaldehyde and 0.1% (v/v) glutaraldehyde buffered at pH 7.2 with 0.1 M PBS buffer. The fixed samples were dehydrated and embedded in LR White resin. Immunolabelling on ultrathin sections were performed with the same procedure as for leaf.

## Authors' contributions

LT and SSMS conceived of the study and participated in its design. LT carried out the experiments. LT and SSMS performed the statistical analysis and prepared the manuscript. All authors read and approved the final manuscript.

## Supplementary Material

Additional file 1 **Impact of total soluble protein extracted from tobacco leaves on the proliferation of NFS-60 cells**. The bioactivities of total soluble protein samples from young, old and freeze-thaw treated leaves in promotion of the proliferation of NFS-60 cells were compared. Click here for file

Additional file 2 **Summary on expression of human colony-stimulating factor (h-CSF) by plant-based platform**. The published reports on the expression of h-CSF in different plants, including transformation method, vector construction, expression level, molecular weight and bioactivity of the target protein, were summarized. Click here for file

Additional file 3 **Dimerization analysis of hG-CSF expressed in transgenic tobacco leaves and seeds**. Immunoblot analysis on non-reduced samples of total soluble protein from leaves and seeds showed that partial dimerization of hG-CSF through disulfide bonding occurred in both SH and USH transgenic seeds while no dimer formed between hG-CSF monomers in leaves. Click here for file

Additional file 4 **Impact of ubiquitin moiety at N-terminus on the expression of hG- CSF in transgenic tobacco**. In this experiment, Construct H carrying the hG-CSF gene and Construct UH containing the ubiquitin and hG-CSF genes, were used for tobacco transformation. As both constructs were without a signal peptide, the synthesis of hG-CSF was directed in the cytosol. Results showed that accumulation of hG-CSF was only detected in UH transgenic plants but not in H plants. Click here for file
