# Supplementary material for: Ubiquitin fusion expression and tissue-dependent targeting of hG-CSF in transgenic tobacco
Source: BMC Biotechnol. 2011 Oct 11;11:91. doi: 10.1186/1472-6750-11-91 (PMC3212944; doi:10.1186/1472-6750-11-91)

Title: Ubiquitin fusion expression and tissue-dependent targeting of hG-CSF in transgenic tobacco (Li Tian and Samuel S.M. Sun)

Additional file 1

Impact of total soluble protein extracted from tobacco leaves on the proliferation of NFS-60 cells. Total soluble protein (TSP) extracted from young (Y), old (O) and freeze-thaw treated (B) leaves was used to treat NFS-60 cells and the bioactivity assay was performed as mentioned in Methods. CT, untreated sample; USH-Y and USH-B, TSP from young and freeze-thaw treated leaves from USH plant containing the expressed hG-CSF (1 ng); USH-B+G, USH-B samples supplemented with additional comercial hG-CSF (1 ng); WT-Y, WT-O and WT-B, TSP from young, old and freeze-thaw treated young leaves of wild type plant containing the same amount of TSP as the USH samples; WT-Y+G, WT-O+G and WT-B+G, WT-Y, WT-O and WT-B samples supplemented with 1 ng comercial hG-CSF. Number in Y axis represented the relative value to untreated sample (CT).

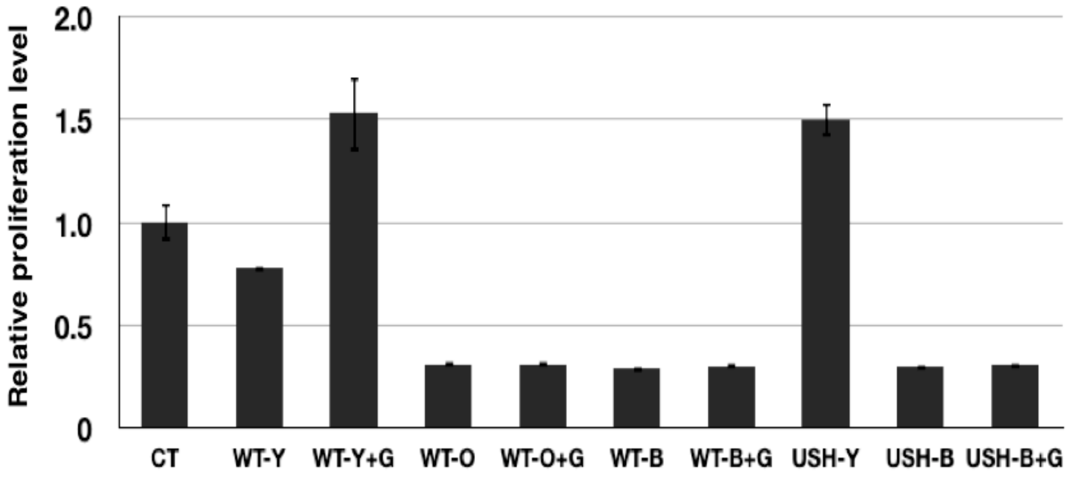

Supplement: Additional file 1 — Impact of total soluble protein extracted from tobacco leaves on the proliferation of NFS-60 cells. The bioactivities of total soluble protein samples from young, old and freeze-thaw treated leaves in promotion of the proliferation of NFS-60 cells were compared. [file 1472-6750-11-91-S1.PDF]
