# Supplementary material for: Ubiquitin fusion expression and tissue-dependent targeting of hG-CSF in transgenic tobacco
Source: BMC Biotechnol. 2011 Oct 11;11:91. doi: 10.1186/1472-6750-11-91 (PMC3212944; doi:10.1186/1472-6750-11-91)
Supplement: Additional file 2 — Summary on expression of human colony-stimulating factor (h-CSF) by plant-based platform. The published reports on the expression of h-CSF in different plants, including transformation method, vector construction, expression level, molecular weight and bioactivity of the target protein, were summarized. [file 1472-6750-11-91-S2.PDF]

Title: Ubiquitin fusion expression and tissue-dependent targeting of hG-CSF in transgenic tobacco (Li Tian and Samuel S.M. Sun)

Additional file 2

Summary on expression of human colony-stimulating factor (h-CSF) by plant-based platform

**h-CSF**	**Plants**	**Transformation method[1]**	**Expression strategy**	**Expression level[2]**	**Molecular weight[3]**	**Bioactive[4]**	**Refs[5]**
hG-CSF	Tobacco cell culture	Agro-T	Binary vector; *CaMV*35S promoter	105 µg/L (9 dpi)	N/A	Yes	[1], 2002
	Rice cell culture	Bom-T	Binary vector; rice α-amylase promoter and signal peptide	185 µg/L (7 dpi)	20 kD (≈E)	Yes	[2], 2006
	Tobacco leaves	T/Agro-I	Viral vector based on the tobacco mosaic virus genome infecting cruciferous plants (crTMV); actin promoter	0.5 mg/g FW (5 dpi)	17 kD (≈E)	Yes	[3], 2009

hGM-CSF	Tobacco cell culture	Agro-T	binary vector; *CaMV* 35S promoter; addition of a tobacco etch virus (TEV) leader sequence to the N-terminus of the GM-CSF gene	150-250 mg/L	14-30 kD	Yes	[4], 2000
	Tobacco cell culture	Agro-T	Binary vector; *CaMV* 35S promoter; addition of stabilizing polymer in culture medium	783 μg/L	N/A	N/A	[5], 2002
	Tobacco seeds	Agro-T	Binary vector; Gt 1 or Gt3 promoter and signal peptides; fusion expression to glutelin	0.03 % TSP	19-21 kD (>E); 50 kD for dimer	Yes	[6], 2002
	Tomato cell culture	Agro-T	Binary vector; *CaMV* 35S promoter	45 μg/L (10 dpi)	N/A	N/A	[7], 2003
	Rice cell culture	Bom-T	Binary vector; rice α-amylase promoter and signal peptide	129 mg/L (5 dpi);	30 kD (>E)	Yes	[8], 2003
	Tobacco cell culture	Agro-T	Binary vector; *CaMV*35S promoter	150.4 μg/L (5 dpi)	N/A	N/A	[9], 2004

Sugarcane leaves	Agro-T	Maize or sugarcane polyubiquitin promoter; potato proteinase inhibitor II signal peptide; retention to ER	0.02%TSP	17-24 kD (>E)	Yes	[10], 2005
Tobacco leaves	T/Vir-I	Potato virus X vector system; *CaMV* 35S promoter	0.2-2.0% TSP	N/A	Yes	[11], 2006
Rice seeds	Agro-T	Binary vector; Gt1 promoter and signal peptide;	1.3% TSP	19-44 kD (>E)	Yes	[12], 2007
Arabidopsis thaliana Seeds	Agro-T	Binary vector; soybean β-conglycinin promoter; phytohemagglutinin signal peptide; modification on 3'-untranslated region of hGM-CSF gene	0.049 % TSP	21 kD (>E)	Yes	[13], 2007
Rice cell culture	Bom-T	Binary vector; rice amylase promoter and signal peptide; RNAi-mediated silencing of cysteine proteinase	289 mg/L (15 dpi)	N/A	N/A	[14], 2008
Rice cell culture	Bom-T	Binary vector; rice amylase promoter and signal peptide; co-expression of proteinase inhibitor	250 mg/L (23 dpi)	N/A	Yes	[15], 2008

Rice cell culture	Bom-T	Binary vector; *CaMV*35S promoter; RNAi-mediated silencing of rice α-amylase	280 mg/L	N/A	N/A	[16], 2008
Tobacco leaves	T/Agro-I	Viral vector based on the tobacco mosaic virus genome infecting cruciferous plants (crTMV); actin promoter	0.3 mg/g FW (5 dpi)	24 kD (>E)	Yes	[3], 2009

[1]: Agro-T, *Agrobacterium*-mediated transformation; Bom-T, Bombardment-mediated transformation; T/Agro-I, Transient expression using *Agrobacterium* injection; T/Vir-I, Transient expression using virus infection.

[2]: TSP, total soluble protein; FW, fresh weight of leaves; dpi, days post-inoculation for cell suspension culture system, or days post-injection for *Agrobacterium*-mediated injection system.

[3]: N/A, molecular weight of the expressed hG-CSF or hGM-CSF was not available; ≈E, molecular weight similar to the recombinant hG-CSF or hGM-CSF produced from *E. Coli*; >E, molecular weight higher than that of the recombinant hG-CSF or hGM-CSF produced from *E. Coli*.

[4]: Bioactivity observed by cell proliferation test; N/A, the bioactivity was not detected.

[5]: cited references and the published year.

References

1. Hong SY, Kwon TH, Lee JH, Jang YS, Yang MS: **Production of biologically active hG-CSF by transgenic plant cell suspension culture**. *Enzyme Microb Tech* 2002, **30**:763-767.

2. Hong SY, Kwon TH, Jang YS, Kim SH, Yang MS: **Production of bioactive human granulocyte-colony stimulating factor in transgenic rice cell suspension cultures**. *Protein Expres Purif* 2006, **47**:68-73.

3. Zvereva AS, Petrovskaya LE, Rodina AV, Frolova OY, Ivanov PA, Shingarova LN, Komarova TV, Dorokhov YL, Dolgikh DA, Kirpichnikov MP*,* Atabekov JG: **Production of biologically active human myelocytokines in plants**. *Biochem (Mosc)* 2009, **74**:1187-1194.

4. James EA, Wang CL, Wang ZP, Reeves R, Shin JH, Magnuson NS, Lee JM: **Production and characterization of biologically active human GM-CSF secreted by genetically modified plant cells**. *Protein Expres Purif* 2000, **19**:131-138.

5. Lee JH, Kim NS, Kwon TH, Jang YS, Yang MS: **Increased production of human granulocyte-macrophage colony stimulating factor (hGM-CSF) by the addition of stabilizing polymer in plant suspension cultures**. *J Biotechnol* 2002, **96**:205-211.

6. Sardana RK, Alli Z, Dudani A, Tackaberry E, Panahi M, Narayanan M, Ganz P, Altosaar I: **Biological activity of human granulocyte-macrophage colony stimulating factor is maintained in a fusion with seed glutelin peptide**. *Transgenic Res* 2002, **11**:521-531.

7. Kwon TH, Kim YS, Lee JH, Yang MS: **Production and secretion of biologically active human granulocyte-macrophage colony stimulating factor in transgenic tomato suspension cultures**. *Biotechnol Letters* 2003, **25**:1571-1574.

8. Shin YJ, Hong SY, Kwon TH, Jang YS, Yang MS: **High level of expression of recombinant human granulocyte-macrophage colony stimulating factor in transgenic rice cell suspension culture**. *Biotechnol Bioeng* 2003, **82**:778-783.

9. Kim YS, Kwon TH, Sik YM: **Direct transfer and expression of human GM-CSF in tobacco suspension cell using Agrobacterium-mediated transfer system**. *Plant Cell Tiss Org* 2004, **78**:133-138.

10. Wang ML, Goldstein C, Su W, Moore PH, Albert HH: **Production of biologically active GM-CSF in sugarcane: a secure biofactory**. *Transgenic Res* 2005, **14**:167-178.

11. Zhu YJ, Zhou FY, Wang ML, Albert HH, Moore PH: **Efficient transient expression of human GM-CSF protein in Nicotiana benthamiana using potato virus X vector**. *Appl Microbiol Biotechnol* 2006, **72**:756-762.

12. Sardana R, Dudani AK, Tackaberry E, Alli Z, Porter S, Rowlandson K, Ganz P, Altosaar I: **Biologically active human GM-CSF produced in the seeds of transgenic rice plants**. *Transgenic Res* 2007, **16**:713-721.

13. Wang B, Ma M, Wu TL: **Aptamers improve the expression of a human granulocyte-macrophage colony stimulating factor in transgenic Arabidopsis thaliana seeds**. *J Plant Biol* 2007, **50**:29-37.

14. Kim NS, Kim TG, Kim OH, Ko EM, Jang YS, Jung ES, Kwon TH, Yang MS: **Improvement of recombinant hGM-CSF production by suppression of cysteine proteinase gene expression using RNA interference in a transgenic rice culture**. *Plant Mol Biol* 2008, **68**:263-275.

15. Kim TG, Lee HJ, Jang YS, Shin YJ, Kwon TH, Yang MS: **Co-expression of proteinase inhibitor enhances recombinant human granulocyte-macrophage colony stimulating factor production in transgenic rice cell suspension culture**. *Protein Expres Purif* 2008, **61**:117-121.

16. Yang MS, Kim NS, Kim TG, Jang YS, Shin YJ, Kwon TH: **Amylase gene silencing by RNA interference improves recombinant hGM-CSF production in rice suspension culture**. *Plant Mol Biol* 2008, **68**:369-377.
