# Supplementary material for: Ubiquitin fusion expression and tissue-dependent targeting of hG-CSF in transgenic tobacco
Source: BMC Biotechnol. 2011 Oct 11;11:91. doi: 10.1186/1472-6750-11-91 (PMC3212944; doi:10.1186/1472-6750-11-91)

Title: Ubiquitin fusion expression and tissue-dependent targeting of hG-CSF in transgenic tobacco (Li Tian and Samuel S.M. Sun)

Additional file 4

Impact of ubiquitin moiety at N-terminus on the expression of hG-CSF in transgenic tobacco. Expression cassettes of Constructs H and UH, both without a signal peptide, were presented in panel A and immunoblot analysis of total soluble protein (20 µg) extracted from transgenic H and UH leaves was shown in panel B. For Construct H, i.e. hG-CSF without the addition of signal peptide and ubiquitin, no visible hG-CSF signal was detected in the total soluble protein by immunoblot (panel B, lanes H). For construct UH, it was hypothesized to show similar expression pattern as Construct H because both of them would direct the synthesis of hG-CSF in cytosol where ubiquitin should be cleaved from the final protein product. Interestingly, different from the H transgenic plants, although in relatively low amounts, hG-CSF was detectable in some UH transgenic plants with the same molecular weight (MW, 18.6 kD) as commercially available hG-CSF produced in *E.coli* (panel B, lanes UH and +), suggesting that ubiquitin moiety at N-terminus may protect the protein from proteolytic attack in transgenic plants. Construct H, hG-CSF chimeric gene without phaseolin signal peptide and ubiquitin sequence; UH, as H but fusion with ubiquitin; RB, right border; LB, left border; *CaMV35S* pro, cauliflower mosaic virus 35S gene promoter; and *NOS* ter, nopaline synthase gene terminator. Lanes 1-3, individual plants and +, positive control (10 ng commercial hG-CSF).

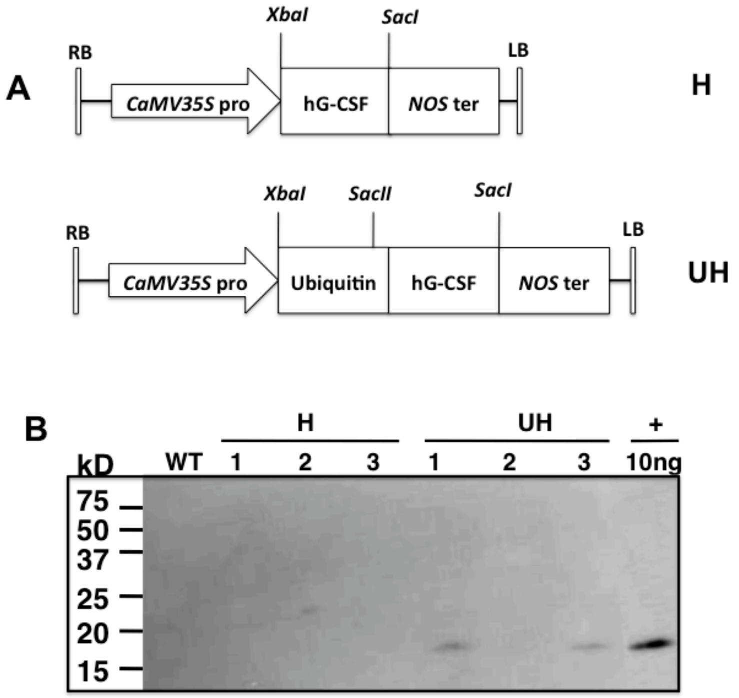

Supplement: Additional file 4 — Impact of ubiquitin moiety at N-terminus on the expression of hG- CSF in transgenic tobacco. In this experiment, Construct H carrying the hG-CSF gene and Construct UH containing the ubiquitin and hG-CSF genes, were used for tobacco transformation. As both constructs were without a signal peptide, the synthesis of hG-CSF was directed in the cytosol. Results showed that accumulation of hG-CSF was only detected in UH transgenic plants but not in H plants. [file 1472-6750-11-91-S4.PDF]
