# Supplementary material for: Ubiquitin fusion expression and tissue-dependent targeting of hG-CSF in transgenic tobacco
Source: BMC Biotechnol. 2011 Oct 11;11:91. doi: 10.1186/1472-6750-11-91 (PMC3212944; doi:10.1186/1472-6750-11-91)

Title: Ubiquitin fusion expression and tissue-dependent targeting of hG-CSF in transgenic tobacco (Li Tian and Samuel S.M. Sun)

Additional file 3

Dimerization analysis of hG-CSF expressed in transgenic tobacco leaves and seeds. To test homo-dimerization of hG-CSF through disulfide bond formation between two free cysteine residues, immunoblot analyses on non-reduced samples of total soluble protein from leaves and seeds, the same protein samples as shown in Figures 2B and 4B, were performed. As shown in the figure below, when total soluble protein of SH and USH leaves was not reduced by β-mercaptoethanol, only a band of the size of monomer was detected (left panel) while no detectable signal of hG-CSF dimer, about 35 kD, as shown by the non-reduced commercial hG-CSF (lane C-), suggesting that no dimer was formed between hG-CSF monomers through disulfide bonding in leaves. For seeds, however, partial dimerization of hG-CSF was observed in both SH and USH transgenic seeds (right panel), suggesting that dimer was formed by two hG-CSF monomers through an interchain disulfide bond and the expressed hG-CSF existed as a mixture of monomers and dimers. WT, USH and SH, total soluble protein from WT, USH and SH plants without treatment of β-mecaptoethanol, respectively; C-, commercial hG-CSF sample without β-mercaptoethanol treatment; and C+, commercial hG-CSF sample treated with β-mercaptoethanol. Arrows denoted dimer of hG-CSF and double-head arrow monomer.

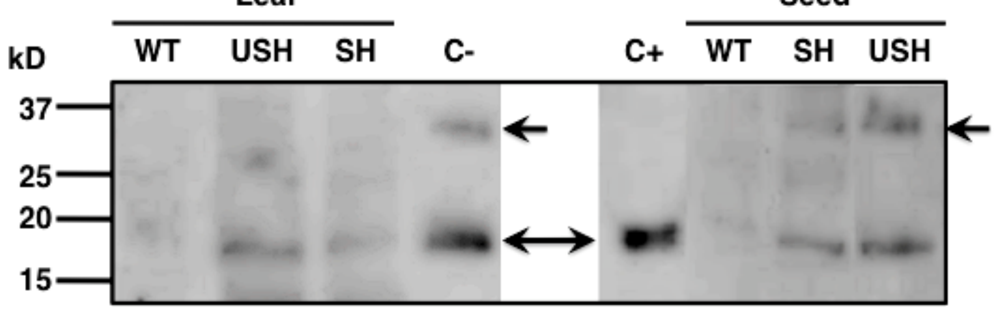

Supplement: Additional file 3 — Dimerization analysis of hG-CSF expressed in transgenic tobacco leaves and seeds. Immunoblot analysis on non-reduced samples of total soluble protein from leaves and seeds showed that partial dimerization of hG-CSF through disulfide bonding occurred in both SH and USH transgenic seeds while no dimer formed between hG-CSF monomers in leaves. [file 1472-6750-11-91-S3.PDF]
